# Evaluating a Pilot Group Based Mental Health Promotion Programme Adapted for Young People With Intellectual Disabilities: The “Healthy Me” Programme

**DOI:** 10.1192/bjo.2022.406

**Published:** 2022-06-20

**Authors:** Emma McDermott, Naomi Jenkins, Heather Hanna, Tammy Morgan

**Affiliations:** Southern Health and Social Care Trust, Northern Ireland, United Kingdom

## Abstract

**Aims:**

A mental health promotion programme called ‘Healthy Me’, was a collaboration between Action Mental Health (AMH) MensSana, Child and Adolescent Mental Health Services (CAMHS) in the Southern Health and Social Care Trust and the Royal College of Psychiatry (RCPsych) in Northern Ireland in 2014. Adapting ‘Healthy Me’ for delivery in special schools was recommended in evaluation of this pilot programme. A co-produced pilot adapted ‘healthy me’ programme, for young people with ID was taken forward by Action Mental Health (AMH) MensSana and Intellectual Disability Child and Adolescent Mental Health Service (ID CAMHS) in the Southern Health and Social Care Trust (SHSCT). To determine the feasibility of adaptation and delivery of the programme for the needs of the ID population. To inform changes to be made before wider roll-out. To promote children's social and emotional well-being and emotional literacy through the teaching of problem-solving, coping skills, conflict management and managing feelings. To evaluate the effectiveness of this intervention with children being able to retain learning, information and ideas.

**Methods:**

Evaluation

- Pre programme quiz July 2021 (young people)
- Post session 1–5 quizzes (young people)
- Post programme quiz October 2021 (young people)
- Simple visual blob tree (young people)

Outcome Measures

- Pre programme initial outcome measure (parent) The Mood, Interest and Pleasure Questionnaire-short form (MIPQ-S) July 2021
- Pre programme initial outcome measure (parent) non standardised, based on the strength and difficulties questionnaire (SDQ) and the Child and Youth Resilience Measure-Revised Person Most Knowledgeable version (PMK-CYRM-R) July 2021
- Post programme repeated outcome measure (parent) MIPQ-S October 2021
- Post programme repeated outcome measure (parent) Based on SDQ & PMK-CYRM-R October 2021

**Results:**

Six participants identified at outset and four attended and engaged consistently, young people aged between 14 and 17 years. Participants were supported 1:1 to fill in a simple evaluation forms after sessions rating their enjoyment and what they had learnt. Repeating the MIPQ-S with parents highlighted some improved scores indicating positive affect and elevated interest and pleasure.

**Conclusion:**

The programme will be offered to in the next stage of the pilot to Special Schools in NI. It is hoped to show that similar positive gains can be made in the school settings for children and young people with intellectual disability in terms of promoting positive mental health and social and emotional well-being.

